# Supplementary material for: Depth-Resolved Macroscopic Fluorescence Lifetime Imaging via High-Spatial-Frequency Structured Illumination
Source: bioRxiv. 2025 Jun 15:2025.06.10.658928. Preprint. [Version 1] doi: 10.1101/2025.06.10.658928 (PMC12259142; doi:10.1101/2025.06.10.658928)
Supplement: Supplement 1 [file media-1.pdf]

Supplementary Information of Depth-Resolved Macroscopic Fluorescence Lifetime Imaging via High Spatial Frequency Structured Illumination

Nanxue Yuan[a,*], Saif Ragab[a], Navid Nizam[a], Vikas Pandey[b], Amit Verma[c], Margarida Barroso[c], Tynan Young[d], John Williams[d], Xavier Intes[a,b]

[a]Department of Biomedical Engineering, Rensselaer Polytechnic Institute, Troy, New York 12180, USA
[b]Center for modeling, simulation and Imaging in Medicine, Rensselaer Polytechnic Institute, Troy, New York 12180, USA
[c]Department of Molecular and Cellular Physiology, Albany Medical College, Albany, New York 12208, USA
[d]City of Hope, Beck Research Institute, Department of Molecular Medicine, Duarte, CA 91016, USA

1 Introduction

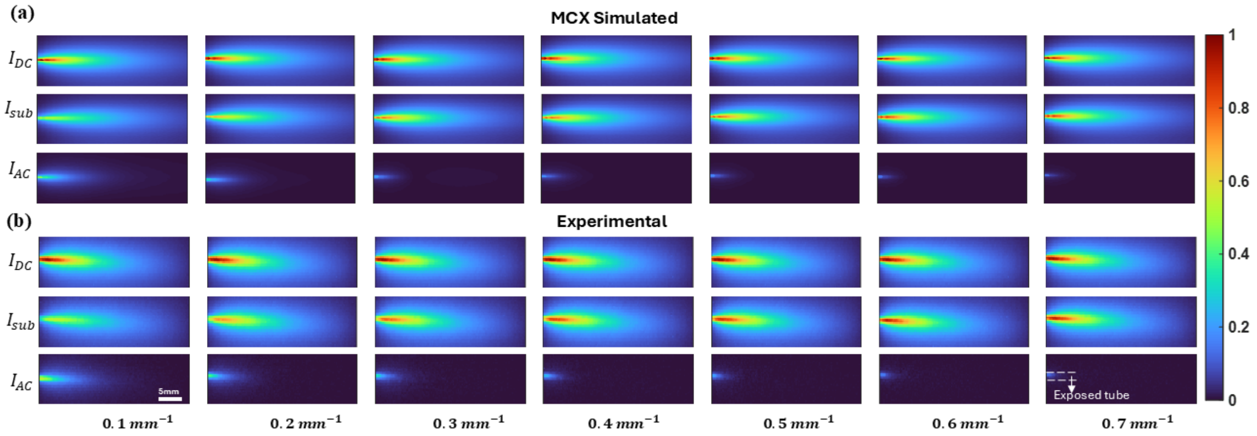

Fig 1 Comparison of fluorescence intensity profiles between MCX simulation (a) and experiments (b). Normalized fluorescence planar signal, I_{DC}, and calibrated subsurface signal,I_{AC}, of a tilted tube at fx = 0.1 to 0.7mm^{-1} in steps of 0.1 mm^{-1}.

	Spatial frequency [mm^{-1}]						
	0.10	0.20	0.30	0.40	0.50	0.60	0.70
I_{AC}	0.993	0.988	0.975	0.953	0.970	0.957	0.926
I_{sub}	0.972	0.982	0.988	0.987	0.989	0.990	0.989

Table 1 R^2 of MXC simulated and Experimental intensity differences at spatial frequency range from 0.1 to 0.7 mm^{-1} by 0.1 mm^{-1}.

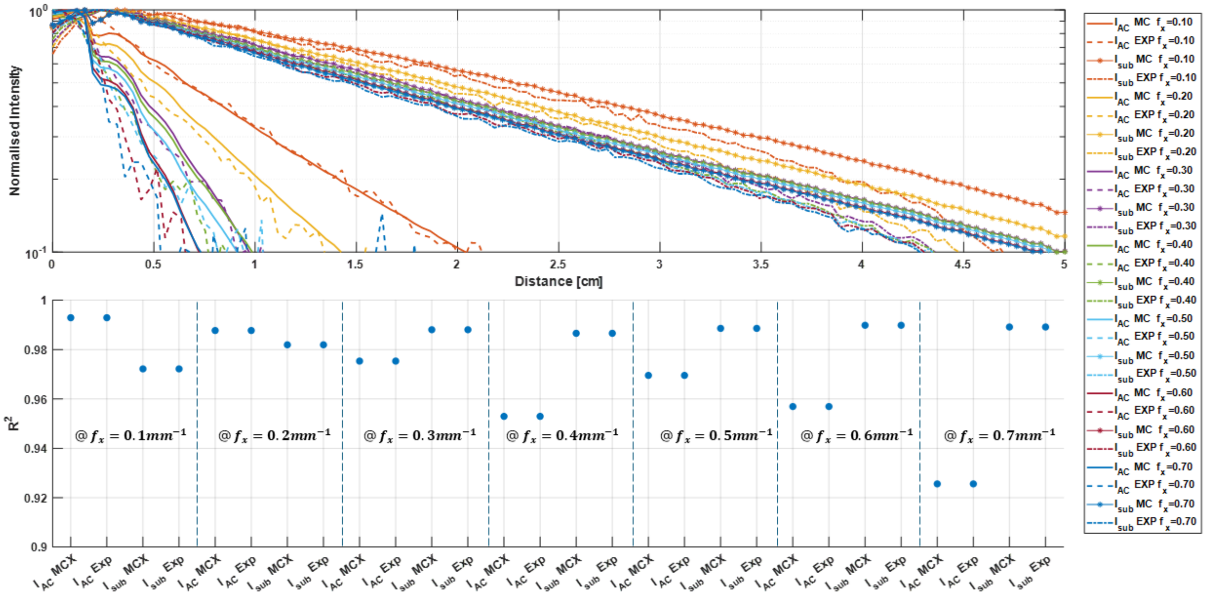

Fig 2 Comparison of normalized Intensity distribution from side A to B follow selected intensity profile in dotted line at fx = 0.1 to $0.7mm^{-1}$ in steps of 0.1 mm^{-1} for (a) MCX simulated and experiment of I_{AC} and I_{sub} separately, (b) corresponding R^2.
